# A Machine Learning Approach for Predicting Pedaling Force Profile in Cycling

**DOI:** 10.3390/s24196440

**Published:** 2024-10-04

**Authors:** Reza Ahmadi, Shahram Rasoulian, Samira Fazeli Veisari, Atousa Parsaei, Hamidreza Heidary, Walter Herzog, Amin Komeili

**Affiliations:** 1 Department of Mechanical and Manufacturing Engineering, University of Calgary, Calgary, AB T2N 1N4, Canada; reza.ahmadi3@ucalgary.ca (R.A.); seyedhamidreza.heida@ucalgary.ca (H.H.); wherzog@ucalgary.ca (W.H.); 2 Human Performance Laboratory, Faculty of Kinesiology, University of Calgary, Calgary, AB T2N 1N4, Canada; shahram.rasoulian@ucalgary.ca; 3 Department of Biomedical Engineering, University of Calgary, Calgary, AB T2N 1N4, Canada; samira.fazeliveisari@ucalgary.ca (S.F.V.); atousa.parsaei@ucalgary.ca (A.P.)

**Keywords:** neural networks, pedal reaction force, radial and mediolateral forces, cycling

## Abstract

Accurate measurement of pedaling kinetics and kinematics is vital for optimizing rehabilitation, exercise training, and understanding musculoskeletal biomechanics. Pedal reaction force, the main external force in cycling, is essential for musculoskeletal modeling and closely correlates with lower-limb muscle activity and joint reaction forces. However, sensor instrumentation like 3-axis pedal force sensors is costly and requires extensive postprocessing. Recent advancements in machine learning (ML), particularly neural network (NN) models, provide promising solutions for kinetic analyses. In this study, an NN model was developed to predict radial and mediolateral forces, providing a low-cost solution to study pedaling biomechanics with stationary cycling ergometers. Fifteen healthy individuals performed a 2 min pedaling task at two different self-selected (58 ± 5 RPM) and higher (72 ± 7 RPM) cadences. Pedal forces were recorded using a 3-axis force system. The dataset included pedal force, crank angle, cadence, power, and participants’ weight and height. The NN model achieved an inter-subject normalized root mean square error (nRMSE) of 0.15 ± 0.02 and 0.26 ± 0.05 for radial and mediolateral forces at high cadence, respectively, and 0.20 ± 0.04 and 0.22 ± 0.04 at self-selected cadence. The NN model’s low computational time suits real-time pedal force predictions, matching the accuracy of previous ML algorithms for estimating ground reaction forces in gait.

## 1. Introduction

Cycling has become one of the most popular recreational activities in recent years [1,2]. Apart from being used as an economical transportation method and recreational activity [3,4], cycling also serves as a valuable tool in rehabilitation programs [5,6,7,8]. Due to improper pedaling techniques and misuse or overuse of muscles, cyclists suffer from various types of injuries, such as hip pain and anterior knee pain [9,10]. Therefore, understanding the kinetics and kinematics of cycling is crucial not only for reducing the risk of injuries but also for enhancing their contributions to clinical and rehabilitation programs.

Over the past two decades, the advent of wearable devices and instrumented sensors has facilitated the collection of extensive kinetics and kinematics data [11,12,13]. Among these sensors, power meters have garnered significant interest from both professional cyclists and recreationally active individuals, who use them to measure and track their exercise performance. The power output value is proportional to the force that is applied perpendicularly with respect to the crank and the pedaling cadence [14]. While the use of power meter sensors has provided significant data for understanding the biomechanics of cycling and developing lower-limb rehabilitation programs, there are significant limitations with models that use power output for analyzing the biomechanics of cycling motion. Advanced 3-axis force sensors integrated into pedals can measure the resultant pedal force [15]; however, their high cost and complex data acquisition systems make them inaccessible to many individuals. A more economical solution would be single-axis force sensors, but they measure only the vertical component of the pedal force. However, knowing the 3-axis pedal forces is crucial for determining the efficiency of the pedaling technique, the distribution of forces across joints, and the overall mechanical load experienced by the musculoskeletal system.

Although human body models have been used to investigate the kinetics and kinematics of cycling, such as the effects of body parameters on pedaling task performance [16], these models typically focus on the tangential force applied on the crank [4,17]. The radial and mediolateral force components are often ignored because they do not directly contribute to the power generated in the cycling motion. However, these forces significantly impact musculoskeletal biomechanics and body physiology, influencing energy consumption, muscle forces, and the internal forces and moments applied to bones and joints [18,19]. Furthermore, knowing all three force components allows for assessing and improving the cycling technique, with the goal of reducing the radial and mediolateral force components and increasing the tangential component to favor the crank moment [20]. Even in the most advanced human body models, assumptions and simplifications are made when representing the resultant pedaling force solely by its tangential component. Bini et al. [21] studied the relative magnitude and profile of the pedaling force components and showed that although the tangential force is the largest force component for much of the power phase of the pedal cycle, at some crank angles, the radial force is larger than the tangential force.

While there are affordable devices available for monitoring power output in cycling, measuring the 3D pedal reaction force requires expensive equipment. The complex postprocessing, high costs, and fragility of these technologies are the greatest challenges to widespread clinical and research applications. Predicting force components without the use of 3D pedal forces would be a major step in facilitating clinical application and enhancing the accuracy of simulation models. Machine learning (ML) models may serve as an appropriate alternative for predicting the pedal force components from more affordable sensors. ML methods have rapidly spread in recent years due to technological advancements in data collection and processing across various industries [22]. ML involves optimizing a performance criterion based on training data or past experiences to uncover hidden patterns or make predictions on new data. Applying ML methods such as regression-based techniques and classification algorithms has demonstrated promising solutions to existing limitations in motion analysis [23,24,25]. At present, neural networks (NNs) are the most used ML model to predict joint kinematics and kinetics from inertial measurement units (IMUs) [26,27,28].

ML has been used for the prediction of kinematics parameters during gait and other physical activities [29,30,31,32,33], while few studies have focused on predicting these parameters during cycling [34,35]. For instance, pre-trained NNs have been used to calculate lower-limb joint angles and the cadence during stationary cycling [36,37]. ML algorithms have also been used to predict lower-limb kinetics during gait [38,39,40] and to enhance the accuracy of predicting ground reaction forces (GRF) during gait. Oh et al. [39] used an NN to calculate joint forces and moments within 20% without the need for costly force plates. Lim et al. [38] used NN and a single IMU near the sacrum to predict lower-limb dynamics during walking with a maximum 12% normalized root mean square error for joint torque predictions. ML models have also been used to predict lower-limb joint kinematics, kinetics, and muscle forces during over-ground walking using IMUs and electromyography [41]. In summary, these methods show great potential for estimating internal and external musculoskeletal loading to enhance rehabilitation and exercise training outcomes.

To the best of our knowledge, there is a lack of study focusing on using ML algorithms to predict kinetics parameters during cycling. Therefore, the present work was aimed at using ML to predict the radial and mediolateral force components of pedaling during cycling. We assessed our ML model’s performance through intra-subject and inter-subject evaluations. Our method has the potential to calculate the inter-segmental resultant forces at the ankle, knee, and hip during cycling, thereby potentially contributing to decisions in the design of rehabilitation and injury prevention programs and the enhancement of cycling performance and efficiency.

## 2. Methodology

**Figure 1:**
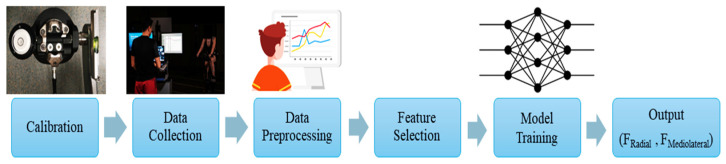
The flowchart for developing the ML model in the present study. After calibrating sensors, data collection was conducted, and the recorded data were preprocessed for feature extraction. An NN was trained to predict radial and mediolateral forces.

The workflow for developing the ML model is illustrated in Figure 1.

### 2.1. Data Collection

Fifteen healthy individuals (10 males, 5 females; age = 29.3 ± 3.6 years; height = 1.71 ± 0.08 m; weight = 73.2 ± 6.9 kg) were recruited for this study at the Human Performance Laboratory at the University of Calgary. Ethics approval was obtained from the University of Calgary Ethics Board (REB #1803), and all participants provided written informed consent before participating in the experiment. Individuals with any neuromuscular or musculoskeletal issues that could affect their cycling ability were excluded from the study. Participants wore tight, minimal clothing and were provided with cycling shoes for their data collection process.

**Figure 2:**
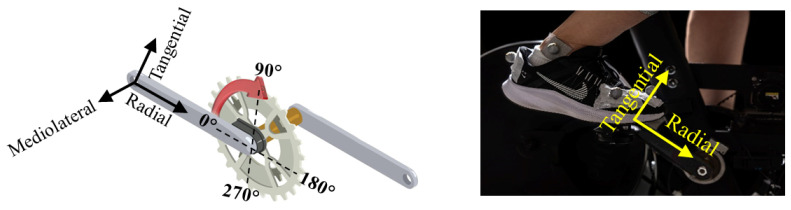
The crank angle was measured from the horizontal position. The radial and tangential forces were measured along and perpendicular to the crank axis, respectively. The mediolateral force was perpendicular to the radial and tangential forces.

A pair of instrumented pedals (ICS-MB, Mountain—BMX, Shimano SPD, available at https://sensix.fr/pedal-sensors_std_22_uk.html (accessed on 4 April 2024)) and an encoder (LEMO FGG.0B.305) were instrumented to the cycling ergometer and controlled by the manufacturer’s software (I-Crankset system, Ver. 4.8.4, SENSIX, Poitiers, France) to collect 3D pedal force data at 250 Hz. The coordinate system of the data acquisition system is illustrated in Figure 2, where radial force was defined along the crank stem, tangential force was defined perpendicular to the crank, and mediolateral force was defined by the vector cross-product of the tangential and radial unit vectors. Standard calibration procedures for force signals were performed as recommended by the manufacturer.

Participants were given cycling shoes with Shimano cleats and asked to warm up by pedaling for 5 min at 100 W at a self-selected pedaling cadence. In addition to preparing the participants for the test, the warm-up trial helped participants familiarize themselves with the device and practice maintaining a constant velocity during the test. The pedaling task protocol comprised two sequential trials, with participants given a rest period between the two trials to eliminate muscle fatigue. The test protocol included two trials at a consistent resistance level but at different cadences: a self-selected cadence (58 ± 5 RPM) and a higher cadence (72 ± 7 RPM), with corresponding power outputs of 96 ± 9 W and 214 ± 17 W, respectively. Each trial lasted 2 min, resulting in approximately 120 to 150 cycles for each trial. To account for pedal rate fluctuations during the pedal cycle, an encoder with a 250 Hz sampling rate was used to pedaling rate and crank angle, similar to the standard cadence sensors commonly found in stationary bicycles. While we grouped pedaling rates into two discrete conditions, namely self-selected and high pedaling rates, the actual rates used by our subjects ranged from 50 to 80 RPM, providing a range that simulates real-world cycling conditions and enhances the accuracy of pedaling force predictions.

### 2.2. Data Preprocessing

The data for each trial were segmented into individual pedaling cycles using a custom labeling function. The first 10 s of data were excluded from analyses to allow participants to reach the target pedaling rate. During intra-subject examinations, “uncleaned” data were used in the training and test phases to simulate real-world conditions. However, for inter-subject examinations, “cleaned” data were used in the training phase to improve model accuracy, while the test phase still employed uncleaned data. In the cleaned dataset, pedaling cycles with total force values falling outside the range of the mean ± 2 standard deviations (SD) during the 2 min test were considered outliers and excluded from the analysis.

### 2.3. ML Development

**Figure 3:**
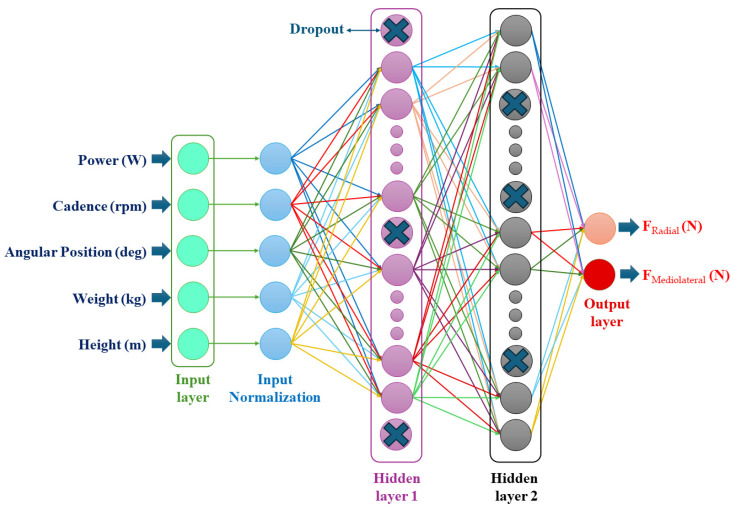
The NN structure consisted of five inputs, including cycling power, cadence, crank angle, and subject weight and height. The output prediction was radial and mediolateral forces.

We used an NN model to predict radial and mediolateral forces from the subject’s body height and weight, power, cadence, and crank angle for each pedaling phase (Figure 3). The MinMaxScaler function from the Scikit-Learn library was employed to scale all variables to the range [0, 1]. It was also necessary to scale the outputs (radial and mediolateral forces) for use with a two-output NN model. The outputs were rescaled back to their original range using the same MinMaxScaler function. The architecture of the model was designed with an input layer consisting of 5 neurons, followed by two hidden layers with 64 and 128 neurons, respectively. The ReLU activation function was applied in each hidden layer. To mitigate the risk of overfitting, a dropout layer with a rate of 0.2 was added after each hidden layer. The output layer consisted of two neurons, corresponding to the two target variables. For the optimization of the NN’s weights, the Adam optimizer was selected with a learning rate of 0.001, and mean squared error was used as the loss function. To enhance the model’s performance, the EarlyStopping function was employed to terminate the training if no improvement was observed for ten consecutive iterations. The batch size was set to 128, and the model was trained for a maximum of 100 iterations. Furthermore, a grid search was conducted to identify the optimal parameters for the model. This included determining the best activation function for the hidden layers from options such as LeakyReLU, ReLU, Sigmoid, GELU, and Tanh. Additionally, the optimal optimizer was chosen from Adam, RMSProp, and SGD, and the ideal learning rate was selected from 0.01, 0.001, and 0.0001. The grid search also assessed the optimal number of neurons in each hidden layer, with options including 64, 128, 256, and 512.

### 2.4. Performance Evaluation

To evaluate the performance of the ML model in predicting forces for individual participants, both intra-subject and inter-subject examinations were conducted. In the intra-subject analyses, 70% of cycles for each subject were used to train the ML model, while the remaining 30% of cycles were used to validate the prediction accuracy [41]. This approach allowed us to assess the model’s accuracy and consistency when applied to data from the same individual, providing a precise measure of intra-subject performance.

To evaluate the model’s generalizability to unseen participants, an inter-subject examination was performed using leave-one-out (LOO) cross-validation. In this approach, the dataset was split into training and testing sets across N iterations (N representing the total number of participants). In each iteration, the model was trained on data from N − 1 participants and tested on the data from the excluded participant. Since real-world data often contain outliers and lack detection procedures due to the absence of force sensors, raw data were used during the evaluation of model performance. This process resulted in 15 distinct training/testing combinations, producing 15 unique ML models.

The root mean square error (RMSE) between the ground truth and the predicted targets was calculated for both intra-subject and inter-subject examinations. RMSE values were reported for each cycle and participant, providing a detailed evaluation of the model’s performance. These RMSEs were averaged across all participants for cross-validation purposes. To better interpret the prediction errors for each force component, the nRMSE was calculated, which is the RMSE normalized to the range of the force data within a cycle. A paired *t*-test statistical analysis was conducted on nRMSE values for the two different cadences: self-selected and high. *p*-values at a 95% confidence level were obtained to determine the significant difference between the mean values of the two groups. Data processing, ML model development, and statistical analyses were conducted in Python 3.12.3.

## 3. Results

We developed an ML method for predicting mediolateral and radial forces from the power level for pedaling tasks at two power intensities. The ground truth pedal force data were measured by SENSIX system with an accuracy range of 1.88 to 2.5 N in the two directions. Predictions were validated through intra-subject and inter-subject comparisons with the actual pedal force measurements. A subject description and pedaling kinematics and kinetics are presented in Table 1. Despite performing a constant-velocity cycling task, subjects could not maintain a consistent power output, leading to variations in power output and cadence parameters, as indicated by the standard deviation in Table 1.

### 3.1. Radial Force

**Figure 4:**
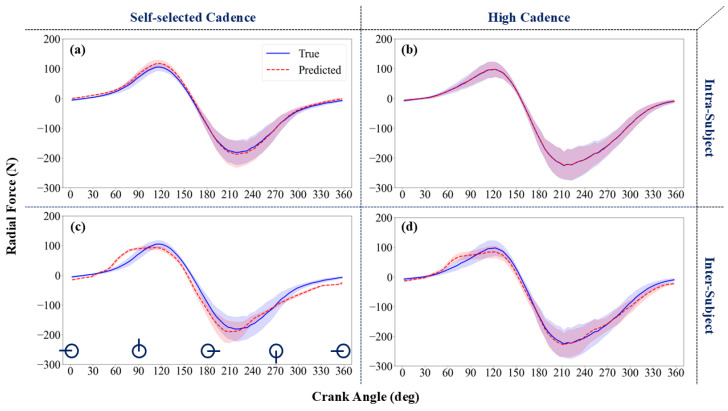
Radial force prediction using the developed ML methods: intra-subject (**a**,**b**) and inter-subject (**c**,**d**) analyses. Radial forces were predicted at self-selected (**a**,**c**) and high (**b**,**d**) cadences from the cross-validation set. A schematic representation of the pedal position is shown next to the *x*-axis in subfigure (**c**). The lines represent the mean values, while the shaded areas indicate the standard deviations.

Figure 4 presents the mean ± SD of the predicted radial force component in the intra-subject (subfigures a and b) and inter-subject (subfigures c and d) analyses. Negative values of radial force, which represent a tensile force applied along the crank, occurred at crank angles of 160 to 350 degrees. While the prediction of radial force in the intra-subject examination had high accuracy across all crank angles, larger errors were observed in the inter-subject examination, especially at crank angles 60–90 degrees during the trial with the higher cadence.

### 3.2. Mediolateral Force

**Figure 5:**
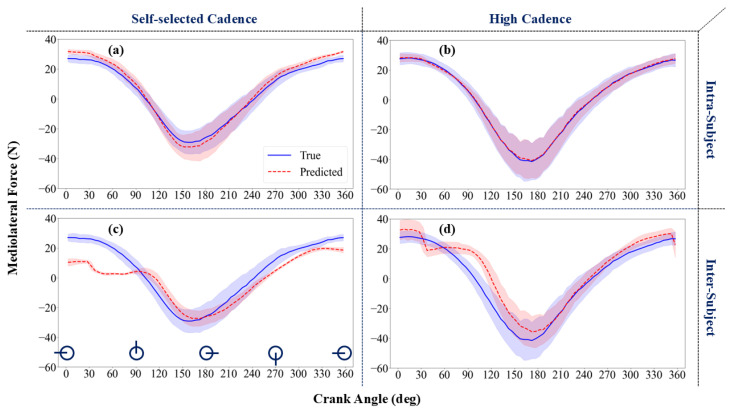
Mediolateral force prediction using the developed ML methods: intra-subject (**a**,**b**) and inter-subject (**c**,**d**) analyses. Mediolateral forces were predicted at self-selected (**a**,**c**) and high (**b**,**d**) cadences from the cross-validation set. A schematic representation of the pedal position is shown next to the x-axis in subfigure (**c**). The lines represent the mean values, while the shaded areas indicate the standard deviations.

Similar to Figure 4, Figure 5 compares the predicted mediolateral force with the measured pedal forces. Prediction accuracy was higher in the intra-subject examination compared to the inter-subject one. The variation in mediolateral force was larger than that of the radial force, as depicted by a relatively higher SD, represented by a thicker highlighted band. Consequently, the mediolateral force prediction accuracy was lower for the trial with the higher cadence, specifically for the pull-up phase, i.e., crank angles 270–360 degrees and 0–90 degrees.

The corresponding RMSE values for the radial force predictions at the high cadence (Figure 4b,d) were 11.0 ± 2.5 N for the intra-subject examination and 33.4 ± 6.8 N for the inter-subject examination (Table 2). For the self-selected cadence (Figure 4a,c), the RMSE values for radial force were 12.1 ± 3.0 N (intra-subject) and 39.2 ± 6.9 N (inter-subject). The RMSE for the mediolateral force predictions were 4.0 ± 0.8 N (intra-subject) and 9.3 ± 2.0 N (inter-subject) for the self-selected cadence (Figure 5a,c), and 3.7 ± 0.7 N (intra-subject) and 9.9 ± 2.4 N (inter-subject) for the high cadence (Figure 5b,d) (Table 2). The difference in nRMSE values between self-selected and high cadence was statistically significant.

## 4. Discussion

**Figure 6:**
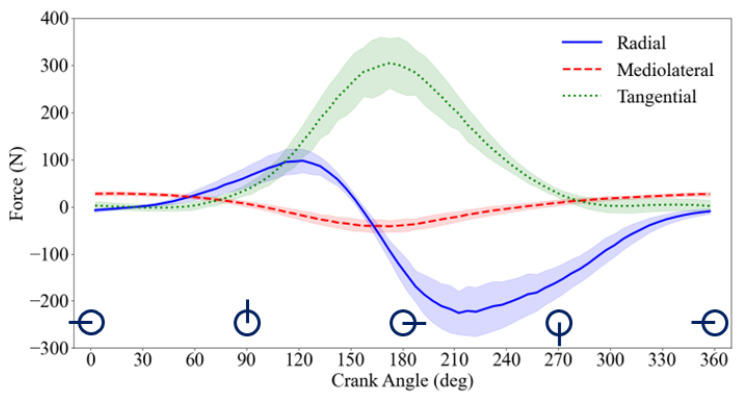
The mean ± SD values for all force components (radial, mediolateral, and tangential) were measured by advanced 3-axis pedal force sensors, highlighting the contribution of each component in resultant pedal reaction force. The lines represent the mean values, while the shaded areas indicate the standard deviations.

In the present study, an ML model was developed for prediction of the radial and mediolateral forces from five pedaling parameters. When tangential, radial, and mediolateral force components are compared for the pedaling task, as shown in Figure 6, the radial force is not negligible compared to the tangential force and cannot be overlooked. For instance, in crank angles between 90 and 270 degrees, the magnitude of the radial force is generally larger than the tangential force, which aligns with the results of previous studies [21,42,43,44,45]. Moreover, the resultant force and its direction play a crucial role in determining pedaling efficiency, force distribution across joints, and musculoskeletal loading conditions. Therefore, in this study, we attempted to propose an accessible approach for prediction of resultant pedaling force.

For this purpose, an NN model was developed to predict radial and mediolateral force components using affordable sensor data and participant characteristics. Five features were selected from a pool of candidates, including lower-limb segment length, weight, height, gender, vertical force, pedal angle, seat distance from the bottom bracket, power, crank angle, and cycling cadence. The final feature set was determined through a trial-and-error process to maximize accuracy. It should be noted that saddle height was set at 109% of the participant’s inseam length, measured from the bottom bracket to the top of the saddle [46]. Thus, only height was included to avoid redundancy in input features and reduce model complexity. Lower-limb segment length was also excluded, as it was closely correlated to the subject’s height. Gender was evaluated as an input parameter but was excluded due to its minimal impact on model performance for predicting pedal force components. The influence of gender may have been inherently captured by the weight feature, as females generally weigh less than males. Future research with larger and more diverse populations may further explore the relevance of gender in cycling dynamics and force prediction.

Two approaches were employed: intra-subject and inter-subject. The intra-subject approach aimed for high accuracy by using data from one session to modify the NN model, facilitating effective monitoring of individuals outside the lab after initial data collection. This approach is ideal for scenarios requiring high precision and feasible initial data collection. For instance, the intra-subject examination for the ML model could be used in rehabilitation programs. In clinical settings, instrumented cycling devices with 3-axis pedal forces measurements might be available and can be used for model training. Subsequently, progress can be monitored using commonly accessible cycling ergometers equipped with power meters as patients continue their rehabilitation programs. For those unable to collect session data, the inter-subject approach offers a viable alternative, enabling predictions without the need for initial data collection.

The RMSE and its normalized form, nRMSE, were averaged across all participants’ data at self-selected and high cadences in the test dataset (Table 2). As expected, the RMSE for the intra-subject examination was lower than that for the inter-subject examination for both radial and mediolateral force components. For radial force, the RMSE values were 39.2 ± 6.9 N and 33.4 ± 6.8 N for self-selected and high cadences, respectively. For mediolateral force, the RMSE values were 9.3 ± 2.0 N and 9.9 ± 2.4 N for self-selected and high cadences, respectively. The reduced accuracy in the inter-subject examination is attributed to the fact that the training dataset and the cross-validation data are not from the same participants. To improve the model’s accuracy in inter-subject examinations, cleaned data were used during the training phase. However, for intra-subject examinations, uncleaned data were used in the training and test phases to simulate real-world conditions, where outliers are common due to the absence of pedal force sensors like those used here for detecting outliers. This indicates that the kinetics of the pedaling task, although comprising a closed-chain and cyclic motion, vary from subject to subject. Such subject-specific mechanical responses can be attributed to differences in joint range of motion, motor control, and pedaling techniques implemented by the participants [47,48]. Testing a larger number of participants across a wider range of power outputs could improve the model’s generalization.

When RMSE was normalized by the range of each force (nRMSE), the prediction error for radial force was consistently lower than for mediolateral forces at both self-selected and high cadences. The lower accuracy in predicting mediolateral force could be attributed to participants’ limited experience in minimizing and controlling this force during cycling, which is sensitive to biomechanical factors such as frame geometry and power output [18]. Additionally, the accuracy of mediolateral forces predictions was notably lower during the pull-up phase of cycling, specifically at crank angles of 270–360 degrees and 0–90 degrees (Figure 5c,d). During this phase, participants did not actively engage their muscles to pull up the crank, leading to greater variability in mediolateral force. The larger relative standard deviation in mediolateral force suggests considerable variability from subject to subject and cycle to cycle despite the lack of degrees of freedom in the mediolateral direction at the pedals.

Our results indicated that the accuracy of predicting radial and mediolateral force components was higher in the high cadence trial compared to the self-selected cadence trial (*p* < 0.05) (Table 2). This improved accuracy at higher cadences may be due to the brain having less time to coordinate muscles for generating non-productive forces such as mediolateral and radial forces. This observation is consistent with studies showing reduced asymmetry in lower-limb functions at higher cadences or power outputs compared to lower cadences [49]. Additionally, research has demonstrated that the gross efficiency of professional road cyclists is higher at elevated power outputs; for example, gross efficiency was greater at 100 RPM compared to 60 RPM [50]. This suggests that, at higher power outputs, the relative contribution of mediolateral and radial force components is reduced compared to the tangential force component.

Since there were no prior studies predicting radial and mediolateral force components for pedaling tasks, we compared the accuracy of our ML model with studies on gait. Our model achieved an average nRMSE of 0.05 ± 0.01 for the radial force predictions in the intra-subject examination and 0.15 ± 0.02 in the inter-subject examination at the self-selected cadence. For mediolateral force prediction, the nRMSE values were 0.13 ± 0.03 in intra-subject and 0.26 ± 0.05 in inter-subject examinations (Table 2). This level of accuracy is comparable to previous studies using different ML algorithms for estimating GRF during gait. For example, peak GRF for ballet jumps was estimated with nRMSE values ranging from 0.17 to 0.38 [51]. Another study used instrumented insoles and regression models to estimate GRF and moments in 5 m straight walking, side-step turn, and cross-step turn, and their model’s nRMSE varied in the range of 0.04–0.2 for different force components [52]. Moghadam et al. evaluated four non-linear regression ML models for estimating lower-limb joint kinematics, kinetics, and muscle forces in walking over-ground using IMUs and EMG data, with nRMSE values for joint kinetics ranging from 0.05 to 0.35 for intra-subject and 0.07 to 0.42 for inter-subject examinations [41]. The NN model in the present work demonstrated potential effectiveness and accuracy in predicting the mediolateral and radial force components of the pedaling task based on power output data. The rapid inference time of the NN model is a notable advantage for applications requiring real-time feedback, such as sports performance monitoring and rehabilitation. However, the model’s performance was lower in predicting the mediolateral force, which is the smallest force component in the pedaling task, particularly at lower cadences. Given the current limitations, future research should focus on further refining the ML model, incorporating more diverse datasets, and evaluating its performance across various cycling protocols to enhance its clinical applicability. Additionally, more complex models such as Transformer-based architectures, CNNs, or LSTM/GRU models may be explored in future studies to capture more complex patterns and dependencies in the data. While these models may improve accuracy, they also come with increased inference times, which may limit their practicality for real-time applications. Balancing model complexity and time response should be studied in future works to optimize accuracy and practical usability in clinical and sport activities settings. Although the proposed AI-based model provides a means to predict resultant pedaling force from power output, it lacks some of the advantages associated with direct measurements of pedaling force.

In this study, we analyzed pedaling at two power levels within the middle range of the population power spectrum. However, pedaling kinematics exhibit inherent variability influenced by factors such as time, gender, age, body features, and power output [53]. Additionally, pedaling kinetics can vary based on technique—such as preferred pedaling style, pedaling in circles, emphasizing the pull during the upstroke, or the push during the downstroke [54]—as well as physical condition (e.g., healthy versus injured), which were not considered in our ML model training. Addressing these limitations could be achieved through a comprehensive study involving a larger sample size and experimental protocols that simulate various cycling conditions and techniques. In this study, cadences ranging from approximately 50 to 80 RPM were used to develop the machine learning model. For future work, we aim to enhance the model’s robustness by testing a broader range of cadences and power levels. This will include incorporating diverse real-world data from professional cyclists and patients with musculoskeletal injuries and/or pain in the lower limb(s). We anticipate that such a comprehensive approach will improve the model’s ability to predict cycling performance and force outputs across a wide range of health conditions, power output, and cycling ability, thereby enhancing its applicability in real-world clinical settings. Integrating wearable devices such as IMUs could enhance the capture of motion kinematics, correlating with kinetic parameters to improve prediction accuracy. Additionally, advancements in AI and statistical methods may offer more precise estimations of resultant forces, suggesting that future research should explore these newer approaches for further model refinement. It is important to note that while there is a relationship between cadence, power, and the tangential force component that contributes to power generation, other force components, such as radial and mediolateral forces, do not consistently correlate with cadence or power. These components may vary across individuals due to biomechanical differences. Although our model accounted for variations in the power-to-cadence ratio, further research is needed to explore a wider range of power and cadence combinations. Future studies should aim to provide a more comprehensive understanding of how pedal forces behave under diverse cycling conditions. The three components of pedal forces are critical for conducting an inverse dynamic analysis, which was not part of this study. While radial and mediolateral forces were predicted, the tangential force can be inferred from available power data and cadence. Future work should focus on developing a full-body model that incorporates all three pedal force components to calculate joint moments accurately. This approach will enhance the analysis of joint forces and improve the understanding of cycling biomechanics.

## 5. Conclusions

Our study demonstrated that an ML model can accurately predict radial and mediolateral force components during cycling using affordable sensor data and participant characteristics. By employing an NN, we identified a feature set that optimized prediction accuracy through a trial-and-error process. The model showed lower accuracy in predicting mediolateral forces than radial forces, which could be improved by increasing the sample size and examining a broader range of power outputs. The low inference times of the NN make it suitable for real-time applications, such as sports biomechanics and rehabilitation programs. Future research can focus on refining the model, incorporating diverse datasets, and validating its performance across various cycling protocols to enhance clinical applicability.

## Figures and Tables

**Table 1:** Subjects’ parameters, pedaling kinematics, and kinetics, including variations in power output and cadence.

Parameters		Mean	SD
Number of Participant, N = 15			
Weight (kg)		73.2	6.9
Height (m)		1.71	0.08
Crank Length (m)		0.17	
Duration of Test (min)	Self-selected cadence	2	
	High cadence	2	
Power (W)	Self-selected cadence	96	9
	High cadence	214	17
Cadence (RPM)	Self-selected cadence	58	5
	High cadence	72	7

**Table 2:** RMSE and nRMSE values for radial and mediolateral force predictions, averaged across all participants for the self-selected and the high cadence trials. * represents a significant difference (*p*-value < 0.05) compared to corresponding values in the self-selected cadence test.

Examination	Force Component	RMSE (N)		nRMSE	
		Self-Selected Cadence	High Cadence	Self-Selected Cadence	High Cadence
Intra-subject	Radial	12.1 ± 3.0	11.0 ± 2.5	0.06 ± 0.02	0.05 ± 0.01 *
	Mediolateral	4.0 ± 0.8	3.7 ± 0.7	0.16 ± 0.03	0.13 ± 0.03 *
Inter-subject	Radial	39.2 ± 6.9	33.4 ± 6.8	0.20 ± 0.04	0.15 ± 0.02 *
	Mediolateral	9.3 ± 2.0	9.9 ± 2.4	0.22 ± 0.04	0.26 ± 0.05 *

## Data Availability

Data are unavailable due to privacy or ethical restrictions.
